# Vulnerability of Polarised Intestinal Porcine Epithelial Cells to Mycotoxin Deoxynivalenol Depends on the Route of Application

**DOI:** 10.1371/journal.pone.0017472

**Published:** 2011-02-25

**Authors:** Anne-Kathrin Diesing, Constanze Nossol, Sven Dänicke, Nicole Walk, Andreas Post, Stefan Kahlert, Hermann-Josef Rothkötter, Jeannette Kluess

**Affiliations:** 1 Medical Faculty, Institute of Anatomy, Otto-von-Guericke University, Magdeburg, Germany; 2 Institute of Animal Nutrition, Federal Research Institute for Animal Health, Braunschweig, Germany; University of Queensland, Australia

## Abstract

**Background and Aims:**

Deoxynivalenol (DON) is a *Fusarium* derived mycotoxin, often occurring on cereals used for human and animal nutrition. The intestine, as prominent barrier for nutritional toxins, has to handle the mycotoxin from the mucosa protected luminal side (apical exposure), as well as already absorbed toxin, reaching the cells from basolateral side via the blood stream. In the present study, the impact of the direction of DON exposure on epithelial cell behaviour and intestinal barrier integrity was elucidated.

**Methods:**

A non-transformed intestinal porcine epithelial cell line (IPEC-J2), cultured in membrane inserts, serving as a polarised *in vitro* model to determine the effects of deoxynivalenol (DON) on cellular viability and tight junction integrity.

**Results:**

Application of DON in concentrations up to 4000 ng/mL for 24, 48 and 72 hours on the basolateral side of membrane cultured polarised IPEC-J2 cells resulted in a breakdown of the integrity of cell connections measured by transepithelial electrical resistance (TEER), as well as a reduced expression of the tight junction proteins ZO-1 and claudin 3. Epithelial cell number decreased and nuclei size was enlarged after 72 h incubation of 4000 ng/mL DON from basolateral. Although necrosis or caspase 3 mediated apoptosis was not detectable after basolateral DON application, cell cycle analysis revealed a significant increase in DNA fragmentation, decrease in G0/G1 phase and slight increase in G2/M phase after 72 hours incubation with DON 2000 ng/mL.

**Conclusions:**

Severity of impact of the mycotoxin deoxynivalenol on the intestinal epithelial barrier is dependent on route of application. The epithelium appears to be rather resistant towards apical (luminal) DON application whereas the same toxin dose from basolateral severely undermines barrier integrity.

## Introduction

Deoxynivalenol (DON), a mycotoxin belonging to type B trichothecenes, is a secondary metabolite of the fungal plant pathogens *Fusarium graminearum* and *F. culmorum* and occurs predominantly on grains, such as wheat, triticale and maize [1]–[4]. It is the most prevalent trichothecene in crop production in Europe and contaminates common cereal-based diets [5].

Deoxynivalenol is implicated in acute and chronic mycotoxicosis in both humans and farm animals [6]. In humans, deoxynivalenol produces emetic effects and is suspected to induce more severe diseases such as alimentary toxic aleukia (ATA) or increased occurrence of oesophageal cancer [7]–[11]. In animals, low DON concentrations induce anorexia and alter immune function, whereas high DON doses induce vomiting, diarrhoea and malabsorption of nutrients [12], [13]. Pigs were identified to be the most susceptible species [14].

Deoxynivalenol enters the body usually via the oral route and subsequently encounters the intestinal epithelial cells, representing the primary target for alimentary intoxication. *In vivo* and *in vitro* experiments report a rapid absorption in the upper gastrointestinal tract (GIT) [15], a decrease in protein synthesis [16], [17] and various transporters, like GLUT, SGLT-1 and amino acid transporters [18]. Furthermore, organs belonging to the immune system (spleen, thymus, bone marrow) appear to be another target of this agent [19], [20].

Pig experiments showed a rapid and nearly complete systemic absorption (91.5±27.4%), with DON appearing already 15 minutes after oral intake in the serum and reaching peak concentrations already after 1.65±0.79 hours [15]. Gastric emptying time (t½) of digesta was estimated at 4.8 hours and at 1.8 hours for DON. This rapid disappearance indicates that DON leaves the stomach most likely with the liquid phase rather than with the solid (digesta) phase. Furthermore, DON recovery in various parts of the porcine gastro-intestinal tract (GIT) showed the upper GIT, i.e. stomach until proximal jejunum, as the most prominent absorption site. In stomach 88.5% of the initial oral DON dose was recovered whereas only 1.5% and 10% could be detected in the upper small intestine and large intestine, respectively [21]. The rather high amounts of DON in the large intestine were attributed to the long digesta retention time in this part of the gut (6–16 hours) and thus to a likely accumulation of the mycotoxin. However, another explanation could be a re-absorption mechanism from the systemic side. Interestingly, effects on intestinal morphology and cell turnover were seen rather for the mid and distal jejunum than for the upper part (Dänicke et al., unpublished data). This leads to the question how this effect could take place when DON was already absorbed and thus could not get in direct contact with the apical (luminal) side of the epithelium? It could be postulated that DON first enters the blood circulation when absorbed in the upper GIT and then re-enters the intestinal lumen, passing through the more distal located intestinal cells from the blood stream via the basolateral side of the cell. *In vitro* trials give evidence for the existence of an active DON transport in the basolateral to apical direction as opposed to simple diffusion from apical to basolateral in the epithelial cell [22]–[24].

The functional repertoire of the intestinal cell differs: the apical side is equipped with the brush border containing a large number of enzyme and transporter complexes such as lactase, sucrase and aminopeptidase that facilitate breakdown and absorption of nutrients from the gut lumen into the blood circulation [25]. On the basolateral side the transporters for nutrients such as amino acids or sugars prevail. Consequently, agents intending to enter the cell might encounter different resistance from the cell depending on the route of entry and thus differing in its impact on the cell.

The intestinal epithelium is characterised by tight and adherent junctions that facilitate cell-cell contact and restrict the paracellular transport to small hydrophilic molecules. Transmembrane and scaffolding proteins such as zonula occludens-1 protein (ZO-1) and different claudins [26] are responsible for the barrier integrity. DON was shown to disturb epithelial cell layer integrity measured by transepithelial electrical resistance in human intestinal CaCo-2 cells [22].

In light of this background it is necessary to investigate the cell response to the application route of exposure to an agent and thus help to understand the underlying mechanisms of such a complex event as mycotoxicosis. Thus we evaluated the effects of DON on (i) cellular viability and (ii) intestinal barrier integrity, using an *in vitro* model of the intestinal porcine epithelial cell line IPEC-J2. The aim of this study was to evaluate whether DON application either from the apical or basolateral cell side affects the barrier function of the intestine more severe.

## Materials and Methods

### Cell culture conditions

The IPEC-J2 cell line is a non-transformed intestinal porcine epithelial cell line continuously maintained in cell culture. Cells were cultured in Dulbecco’s modified eagle medium (DMEM/Ham’s F-12 [1∶1]) supplemented with 5% foetal calf serum (FCS), 1% insulin-transferrin-selenium (ITS), 16 mM HEPES (all PAN-Biotech, Germany) and 5 ng/mL epidermal growth factor (EGF; BD Biosciences, Germany) incubated at an atmosphere of 5% CO_2_ at 39 °C [27]. Cell cultures were regularly tested by PCR (Venor® GeM Mycoplasma Detection Kit; Minerva Biolabs, Germany) and found to be free of mycoplasma contamination. Cells were routinely seeded at a density of 0.5×10^5^/mL in plastic tissue culture flasks (75 cm^2^ Nunc, Denmark) and passaged every 3–4 days for a maximum of 20 times (passages 70–90). In our experiments, IPEC-J2 cells were grown on non-coated, 1 µm pore-sized culture inserts (6-, 12-, or 24-well ThinCert™, Greiner bio-one, Germany) at a density of 1.0×10^5^/well for 12 and 24-wells and 2.0×10^5^/well for 6-well inserts, respectively, until reaching confluence. By using culture inserts, cells were grown in a two-compartment chamber allowing access from both sides, with an upper compartment subsequently referred to as apical side and a lower compartment referred to as basolateral side. Culture medium, containing increasing concentrations (200, 500, 2000 or 4000 ng/mL) of DON (D0156; Sigma-Aldrich, Germany), was added either in the apical or basolateral compartment for 24, 48 and 72 hours. Assays were performed in singles in at least three independent experiments.

### Preparation of deoxynivalenol (DON)

Purified DON (D0156; Sigma-Aldrich, Germany) was diluted in absolute ethanol (99.6%; Roth, Germany) to prepare a 0.2 mg/mL stock solution. Working solutions were prepared in cell culture medium. A final concentration of 1% ethanol corresponding to the ethanol concentration of 2000 ng/mL DON solution was tested in all assays and results were not significantly different from control.

### Assays in 24-well format

#### Transepithelial electrical resistance (TEER)

Measurement of TEER was performed using a Millicell electrical resistance system (Millipore, France). Cells were deemed to be confluent at a TEER value of ≥1 kOhm/well, which was routinely after 4 days. This value derived from previous trials determining the TEER kinetics of IPEC-J2 cell line in inserts during 21 days (data not shown).

#### Lactate dehydrogenase activity (LDH)

Cellular membrane integrity was assessed by measurement of medium LDH activity (Cytotoxicity Detection Kit, Roche, Germany). LDH assay was performed according to manufacturer’s protocol using 100 µl supernatant of the apical side at different time points (24, 48, 72 h).

#### Immunohistochemical determination of DNA synthesis

DNA-synthesis was detected by 5’-brome-2’-deoxyuridine (BrdU; Roche, Germany) incorporation during the last 6 h of incubation. Membranes were fixed in absolute ethanol at 4°C for 30 min and in acetone for 3 min, detached, washed in Tris-buffered saline (TBS, pH 7.6) with 0.05% Tween and blocked with 1% normal goat serum (NGS, Axxora, Germany) for 10 min. In general, after each individual step membranes were washed in TBS/Tween. Membranes were treated with 2 M HCl at 37 °C for 30 min and neutralised at room temperature in 0.1 M sodium borate (pH 8.5) twice for 5 min. After 30 min at 4 °C in TBS/Tween (0.05%) membranes were incubated with a mouse monoclonal antibody against BrdU (1∶100; BD Pharmingen, USA) for 30 min. A secondary biotin-labelled goat anti-mouse IgG1 antibody (1∶50; Southern biotech, USA) was added for 30 min and thereafter an ABC-reagent (ABC Elite, Vector Laboratories, USA) for 60 min at room temperature. Diaminobenzidine solution (DAB, in 0.1 M PBS, freshly added 0.015% H_2_O_2_) was added for 1 min and membranes were subsequently counter-stained with haematoxylin. The immuno-labelled (brown) and haematoxylin-stained nuclei (blue) were counted manually.

#### Cell count

Detached membranes were fixed in absolute ethanol and acetone. Cells were washed three times with phosphate buffered saline (PBS, pH 7.4 and nuclei stained with the DNA-intercalating fluorescent dye 4’,6-diamidino-2-phenylindole (DAPI, Partec, Germany). Fluorescence microscopy and photographs were performed using an Axiovert 200 M (Zeiss, Germany) equipped with an AxioCam MRm camera and corresponding Axiovision software. DAPI-stained nuclei were counted manually and their respective nucleus area was measured using Axiovision software.

### Assays in 12-well format

#### Analysis of tight junction structure using immunofluorescence

DON concentration used for immunofofluorescence was 2000 ng/mL. Membranes were detached from the culture insert and fixed in absolute ethanol and subsequently in acetone. Cells were washed with phosphate-buffered saline (PBS, pH 7.4) and blocked for 10 min with 1% NGS. Rabbit anti-ZO-1 or rabbit anti-claudin-3, both diluted at 1∶100 (Invitrogen, Germany), were applied as primary and Alexa fluor 488 goat anti-rabbit (green), diluted at 1∶200 (Invitrogen, Germany) as secondary antibody. Nuclei were stained with DAPI (Partec, Germany). Fluorescence microscopy and photographs were done using an Axiovert 200 M (Zeiss, Germany) with an AxioCam MRm camera and corresponding Axiovision software.

### Assays in 6-well format

#### Protein isolation and immunoblotting of ZO-1, claudin-3 and caspase 3

DON concentrations used for immunoblotting comprised 200 and 2000 ng/mL and times were set at 6 and 24 hours for caspase 3. ZO-1 blotting was done for 2000 ng/mL DON at 24, 48 and 72 hours. Cell homogenate protein was obtained by 10 min incubation on ice with SDS-gel loading buffer (1 M Tris base pH 6.8; 1% Glycerol, 10% SDS, 0.1% Bromophenol blue; freshly added with 0.05% β-mercaptoethanol and 1% protease inhibitors; Complete, Roche, Germany) and cells were collected with a cell scraper. Samples were denatured at 95 °C for 5 min and loaded together with a prestained protein ladder (SM1811; Fermentas, Germany) onto 10% SDS-polyacrylamide gels. After electrophoresis and semi-dry blotting onto 0.45 µm nitrocellulose membranes (Whatman, Germany) the primary rabbit anti-ZO-1 antibody (1∶500; Invitrogen, Germany), rabbit anti-caspase 3 antibody (1∶1000, Cell Signaling, Germany) or rabbit anti-claudin-3 antibody (1∶1000; Invitrogen, Germany) were used in blocking reagent of the corresponding detection kit. Detection of primary antibody binding on western blot was done with BM Chemiluminescence Western Blotting Kit mouse/rabbit (Roche, Germany). After ZO-1, caspase 3 and claudin-3 development, the blots were stripped at 50 °C for 30 min with stripping buffer (7.58 g Tris base, 20 g SDS, 7 mL ß-mercaptoethanol, pH 6.8), washed and reprobed with anti-GAPDH antibody (1∶1000, Cell Signaling, Germany). Blots were analyzed on an Alpha-Ease® FC Imaging System (Alpha Innotech, Canada).

#### Cell cycle analysis by flow cytometry

After reaching confluence cells were synchronised for 24 hours in serum free media before being exposed to 2000 ng/mL DON. Cells were trypsinised, pelleted and resuspended in PBS. Ethanol fixation and propidium iodide (PI; Sigma, Germany) staining proceeding were performed as previously described [28]. Cells were analysed on a FACSCalibur flow cytometer using CellQuest Pro® software (both BD Biosciences, Germany).

### Statistical analysis

Data were analysed by ANOVA and *P* values calculated using Dunnett’s or Tukey’s post hoc test (GraphPad Prism 3.0, GraphPad Software Inc.). Each value represents a single measurement of at least three independent experiments. Data are expressed as means (±SEM). Significant differences between treated cells and control are indicated by asterisks * p≤0.05; ** p≤0.01; *** p≤0.001.

## Results

### DON influenced cell number and nucleus area

**Figure 1:**
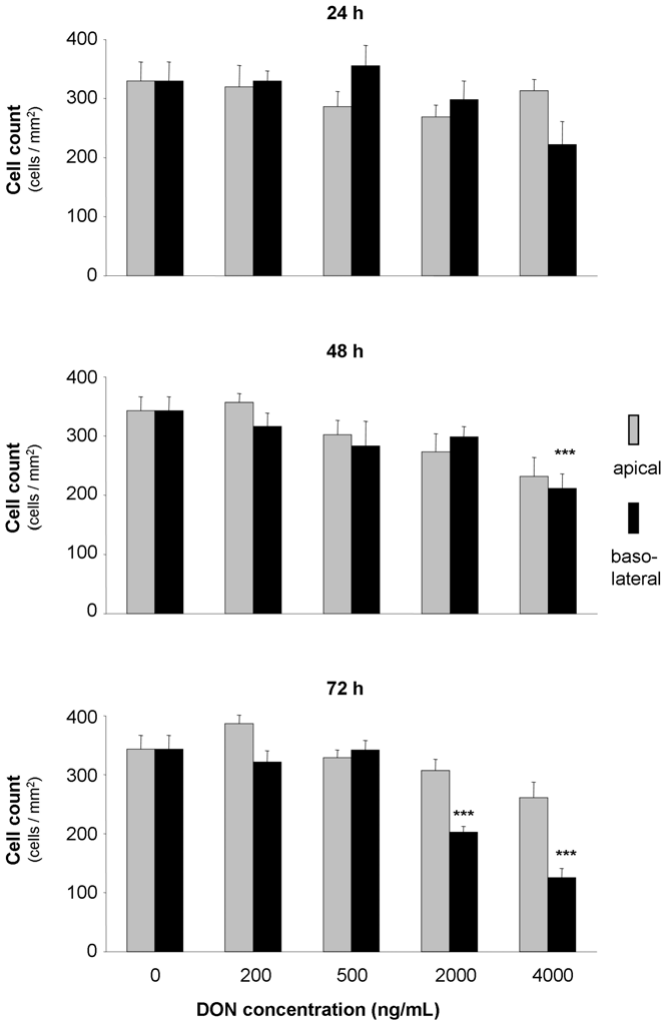
Effect of deoxynivalenol (DON) on total cell count of IPEC-J2. Cells were grown on inserts and incubated for 24, 48 or 74 hours with DON (0–4000 ng/mL) applied from apical or basolateral side in complete medium. Data are given as means (±SEM) in triplicates from three separate experiments. ***p≤0.001 vs. DON0.

The cell number, defined by count of DAPI stained nuclei on membranes, as well as the measured nuclei area, were not influenced by the application of DON from apical side at any concentration or time in comparison to the control (DON0). In contrast, after 24 hours incubation from the basolateral side, we observed a numerical decrease in number of DAPI-stained nuclei at 4000 ng/mL DON. However, this decrease reached statistical significance after 48 hours of DON exposure. After DON incubation of 72 hours we detected a significantly lower cell count at 2000 ng/mL and 4000 ng/mL (Fig. 1). Furthermore we determined the DAPI stained nucleic area. It is noteworthy that we detected a significant enlargement in nucleus area for 4000 ng/mL DON from basolateral for 72 hours incubation only (Tab. 1).

**Table 1:** Deoxynivalenol (DON) enlarged nucleic area (µm^2^) of IPEC-J2 cells.

DON (ng/mL)	24 h		48 h		72 h	
	ap	bl	ap	bl	ap	bl
0	139±10		134±6		137±4	
200	148±17	129±5	133±4	138±16	130±7	130±6
500	138±6	127±17	148±12	148±16	140±14	134±13
2000	144±7	138±6	150±15	143±14	136±9	170±15
4000	144±5	165±25	151±17	163±20	134±6	225±19***

xCells were incubated for 24, 48 or 74 hours with DON (0–4000 ng/mL) in complete medium applied from apical (ap) or basolateral (bl) side. Data are given as means (±SEM) from three separate experiments.

***p≤0.001 vs control.

### DON induced cell proliferation

**Figure 2:**
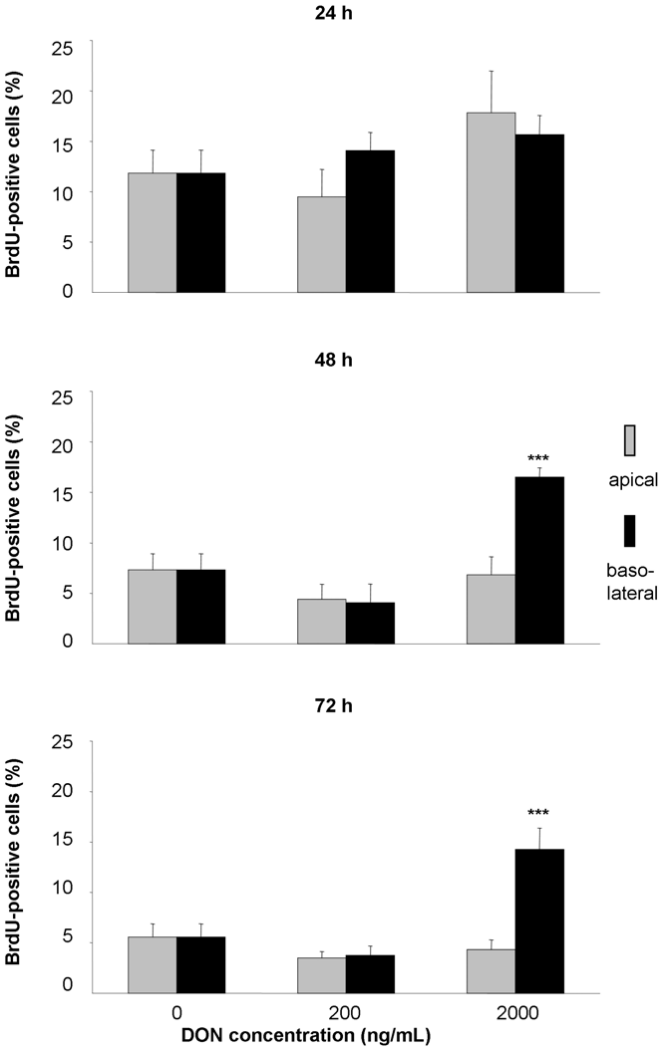
Influence of deoxynivalenol (DON) on proliferation of IPEC-J2 cells. Cells were grown on inserts, synchronised for 24 hours in serum free medium and subsequently incubated for 24, 48 or 74 hours with DON (0, 200 or 2000 ng/mL) applied from apical or basolateral side in complete medium. DNA synthesis was assessed by BrdU incorporation. Data are given as means (±SEM) from four separate experiments. ***p≤0.001 vs. DON0.

DNA-synthesis and thus cell proliferation was measured by BrdU incorporation. We detected a significant relative increase of proliferation after 48 and 72 h incubation with 2000 ng/mL DON from basolateral, whereas apical toxin application showed no effect on DNA synthesis (Fig. 2).

### DON did not affect LDH release

Although cell count did change markedly dependent on route of application of DON we could not observe any alteration in LDH release at any concentration or incubation time when expressed relative to control (data not shown).

### DON did not induce caspase 3 mediated apoptosis

**Figure 3:**
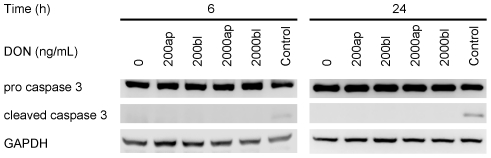
Effect of deoxynivalenol (DON) on apoptosis of IPEC-J2 cells. Cells were grown on inserts and incubated for 24, 48 or 74 hours with DON (0, 200 or 2000 ng/mL) applied from apical or basolateral side in complete medium. Protein expression of pro caspase 3 (35 kDa) cleaved caspase 3 (17 kDa) was analysed by immunoblotting. The housekeeping protein GAPDH (37 kDa) was used as loading control. Staurosporine (100 µM) was used as positive control for cleaved caspase 3.

The protein expression of activated caspase 3, one marker of cellular apoptosis, was determined using immunoblotting. We did not see any appearance of the active cleaved caspase 3 protein in response to DON exposure independent of time, route or concentration (Fig. 3). The protein expression of the housekeeping glycolytic protein GAPDH remained stable under all concentrations or times and staurosporine (100 µM) induced detectable caspase 3 activation.

### DON stimulated DNA-fragmentation and G2/M arrest (Cell cycle analysis)

**Figure 4:**
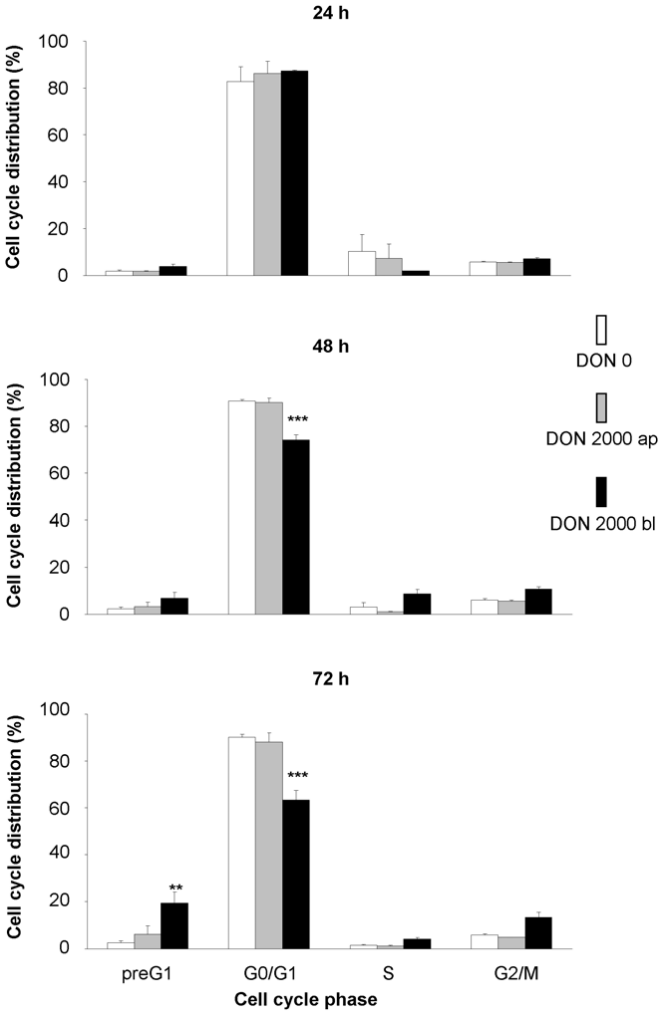
Cell cycle analysis of IPEC-J2 cells treated with deoxynivalenol (DON). Cells were grown on inserts and synchronised for 24 hours in serum free medium and then incubated for 24, 48 or 74 hours with DON (0 or 2000 ng/mL) applied from apical or basolateral side in complete medium. After staining with propidium iodide DNA content was analysed by FACS. Data given are means (±SEM) from five separate experiments. **p≤0.01 vs. DON0, ***p≤0.001 vs. DON0.

Analysis of the cell cycle phases by PI-staining using flow cytometry revealed a marked role of the route of DON exposure on the toxin effect. Apical toxin exposure did not alter the DNA content in comparison to control. Cell exposure to DON 2000 ng/mL from basolateral resulted in a significant decrease in the G0/G1 phase after 48 and 72 hours as well as a significant increase in preG1 phase after 72 hours (Fig. 4). Although we observed a numerical increase in cells in G2/M phase after 72 hour DON-treatment this finding was not statistically significant.

### DON induced breakdown of intestinal barrier integrity

**Figure 5:**
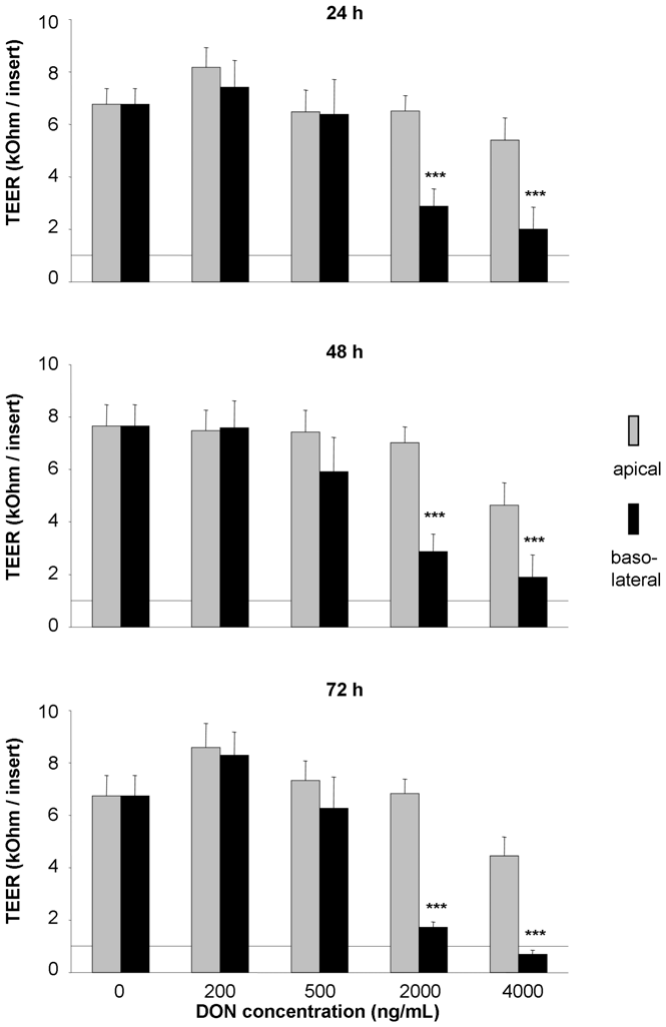
Impact of deoxynivalenol (DON) on transepithelial electrical resistance (TEER) in polarised IPEC-J2 layers. Cells were grown on inserts and incubated for 24, 48 or 72 hours with DON (0–4000 ng/mL) applied from apical or basolateral side in complete medium. TEER values are expressed in kOhms per insert (0.3 cm^2^) with 1 kOhm being the level of confluence. Data are given as means (±SEM) from at least 14 separate experiments. ***p≤0.001 vs. DON0.

TEER is an indicator for the tight junction integrity of a confluent epithelial cell layer. In control cells TEER was essentially constant throughout the experimental period. In our trials with apical DON exposure we did not see a significant change in TEER even with highest concentrations (4000 ng/mL) applied. It is noteworthy that DON added to the basolateral side showed a significant decrease in TEER at 2000 ng/mL and 4000 ng/mL DON already after 24 hours. TEER did not recover throughout prolonged incubation times (Fig. 5).

### DON influenced expression of tight junction proteins

**Figure 6:**
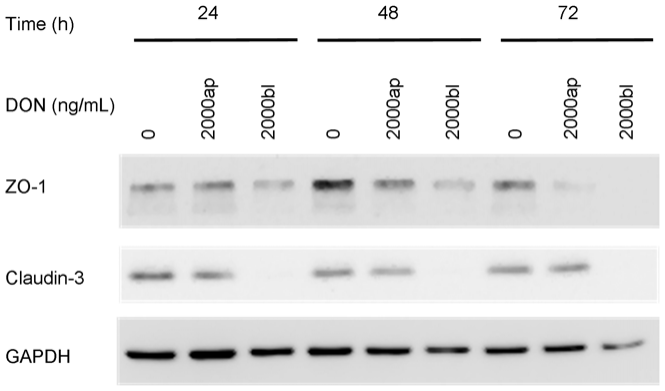
Western blot of tight junction proteins ZO-1 and claudin-3 in IPEC-J2 cells treated with deoxynivalenol (DON). Cells were grown on inserts and incubated for 24, 48 or 74 hours with DON (0 or 2000 ng/mL) applied from apical or basolateral side in complete medium. ZO-1 (225 kDa) and claudin-3 (22 kDa) expression was analysed by immunoblotting. The housekeeping protein GAPDH (37 kDa) was used as loading control.

**Figure 7:**
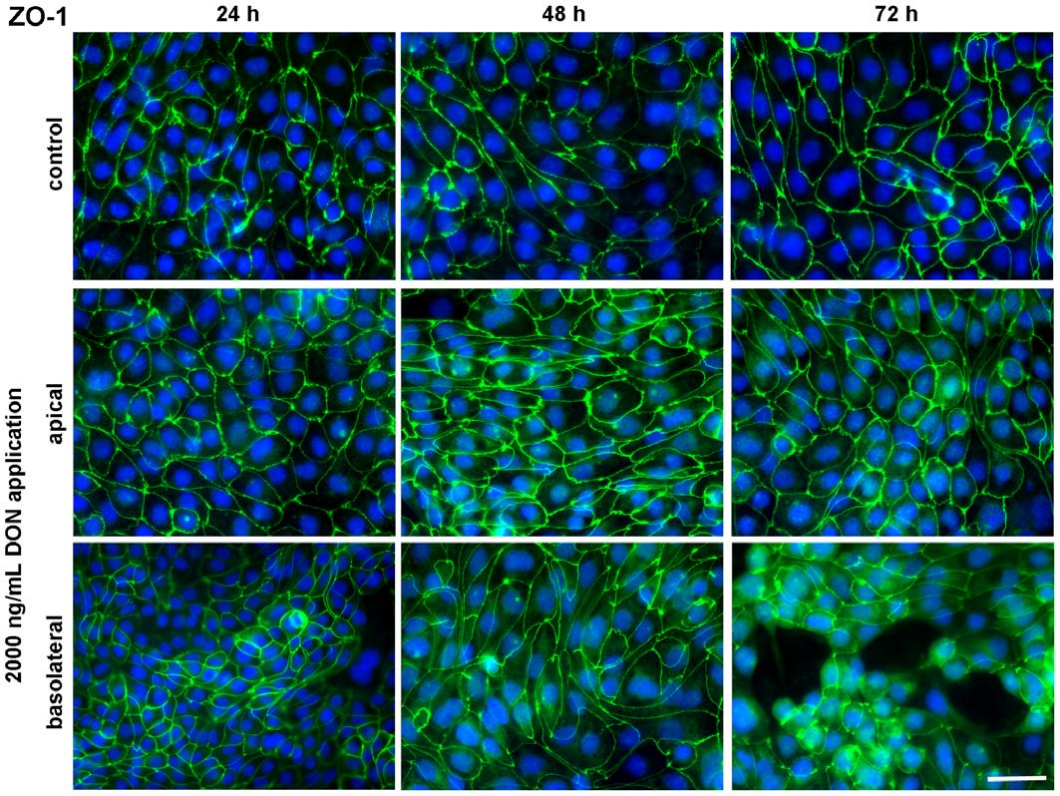
Cellular distribution of the tight junction protein ZO-1 in IPEC-J2 monolayers treated with deoxynivalenol (DON). Cells were grown on inserts and incubated for 24, 48 or 74 hours either without DON (upper panel) or with 2000 ng/mL DON applied from apical (middle panel) or basolateral side (lower panel) in complete medium. Monolayers were stained for the tight junction associated protein ZO-1 and nuclei stained with DAPI, then detected by immunofluorescence microscopy. All micrographs were taken under identical exposure time and in the centre of each membrane. Micrographs are representative for 3 separate experiments with similar results. Scale bar  =  50 µm.

**Figure 8:**
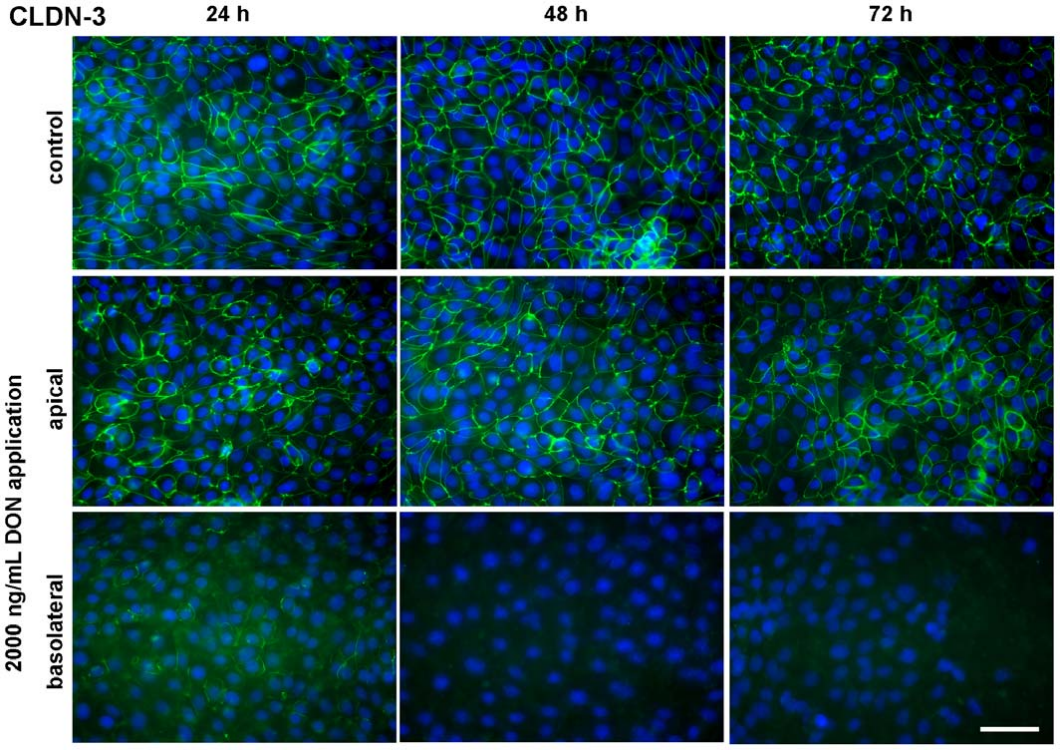
Cellular distribution of the tight junction protein claudin-3 (CLDN-3) in IPEC-J2 monolayers treated with deoxynivalenol (DON). Cells were grown on inserts and incubated for 24, 48 or 74 hours either without DON (upper panel) or with 2000 ng/mL DON applied from apical (middle panel) or basolateral side (lower panel) in complete medium. Monolayers were stained for the tight junction associated protein claudin-3 and nuclei stained with DAPI, then detected by immunofluorescence microscopy. All micrographs were taken under identical exposure time and in the centre of each membrane. Micrographs are representative for 3 separate experiments with similar results. Scale bar  =  50 µm.

The expression of ZO-1 and claudin-3, proteins of the tight junction complex between adjacent cells, was investigated using immunoblotting and immunofluorescence. We found a slight difference after 48 hours between 2000 ng/mL DON from apical or basolateral side, the latter resulting in a lower protein expression. This difference was markedly enhanced after 72 hours incubation, i.e. the ZO-1 expression in cells treated with 2000 ng/mL DON from basolateral disappeared completely from the blot (Fig. 6). In contrast to these findings, the immunofluorescence staining of ZO-1 did not change the distribution pattern between apical or basolateral treated cells with 2000 ng/mL DON at any investigated time point (Fig. 7). ZO-1 was detected as a continuous lining around each epithelial cell independent of application route. However, at 72 hours incubation of 2000 ng/mL DON from basolateral the confluent cell layer was disturbed and thus only cell islets were distributed on the membrane. Interestingly, DON showed a more pronounced impact on claudin-3 protein expressions (Fig. 6) and structure (Fig. 8). Immunoblotting clearly demonstrated the absence of a claudin-3 signal when DON was applied from basolateral at each time point. This effect was absent at apical application. This was confirmed by the immunofluorescence staining, where claudin-3 appeared as a continuous lining around each cell in controls and in cells treated with DON from apical. Basolateral application elicited a definite disturbance of the continuous lining, beginning at 24 h and resulting in a complete disappearance oat 48 and 72 h.

## Discussion

One of the most important functions of the intestinal epithelial cell layer is to form an effective barrier against the uptake of nutritional antigens, pathogens and toxins [29]. Intestinal epithelial cells form a polarised layer which effectively separates the apical (luminal) from the basolateral compartment, i.e. the *lamina propria*. Tight junctions between adjacent cells represent an integral part of this compartmentalisation and any damage to them leads to an enhanced permeability of the cell layer and a decreased TEER which can lead to intestinal disorders.

*In vitro*, cells grown on permeable filter supports are morphologically different from cells grown on non-permeable dishes. They exhibit a more columnar cell type which is represented in an increased layer thickness and a lower contact area to the support (Nossol et al., unpublished data). This morphology is more comparable to the *in vivo* situation where the intestinal barrier is a simple, columnar epithelium [30]. In general, an epithelial cell layer is characterised by its polarity and the generation of two compartments, apical and basolateral, thereby creating a physical barrier. This is facilitated by cell-cell contact and cell-substrate contact [31]. More importantly, *in vivo* the intestinal epithelial cell receives its nutrients from the basolateral as well as from apical side. Cells cultured on non-permeable supports cannot form the two compartments and may not be fully polarised. Additionally, cells have to rearrange their apical domain to accommodate nutrient supply from the apical side solely [32]. This also implies that any agent being exposed to the basolateral side of the epithelial cell also might interfere with the cell’s nutrient supply in some way. This emphasises the importance of the cell support chosen for *in vitro* experiments investigating epithelial or endothelial cell lines.

In our study we observed a significant impact of the mycotoxin DON on intestinal barrier integrity as well as cell viability in dependence on the application route. First, TEER was detrimentally affected already after 24 hours when exposed to 2000 ng/mL DON from basolateral that persisted after 48 and 72 hours toxin exposure. The highest DON level (4000 ng/mL) resulted in a TEER-decrease even below the level of cellular confluence, i.e. 1 kOhm, after 72 hours exposure. It is noteworthy that any apical application of the toxin did not alter TEER compared to the control. Other studies [33], [34] reported also an impact of DON on TEER using IPEC-1, CaCo-2 and HT-29 cells, representing frequently used porcine and human intestinal epithelial cell lines. In these studies the toxin elicited a significant response from the apical side as opposed to our data, but a decrease below the level of confluence was only achieved at the distinctly higher concentrations of 50 and 100 µM (approx. 15 and 30 µg/mL) after 72 hours DON exposure. The basolateral administration was not tested by these authors.

Further indicators for epithelial layer integrity are the scaffolding protein ZO-1 and the transmembrane protein claudin-3, both part of the tight junction complex. They facilitate, besides others, cell-cell contact and the distinction between apical and basolateral compartment. Furthermore, ZO-1 as a scaffolding protein is essential for the spatial organisation of claudins which are primarily responsible for maintenance of TEER [35]. Immunoblotting of ZO-1 showed a fading signal after 48 hours that literally became annihilated after 72 hours when cells were exposed to the toxin from basolateral. Even more pronounced was the impact on claudin-3, which showed a very faint band after 24 h basolateral toxin exposure and complete absence of signals after 48 and 72 h basolateral application. Apical toxin exposure did not change the protein expression as compared to control. This was also reflected in the immunofluorescence of claudin-3. Moreover, the intestinal barrier function, as characterised by the coinciding TEER measurements, was detrimentally affected by the loss of claudin-3. The protein structure of ZO-1 appeared surprisingly intact as seen by immunofluorescence, but viewing the entire cell layer revealed great gaps in the layer, accounting for cell death and disintegration of the epithelium. This also explains the drop in protein amount shown by immunoblotting: although ZO-1 structure between two adjacent cells remained intact, cell losses increased significantly and thus amount of ZO-1 protein diminished on a large scale. Other authors also reported no effect of DON on ZO-1 after 48 h apical incubation despite high doses (30 µM≈9 µg/mL) but a marked deterioration of claudin-3 in the same study [34]. Total cell count dropped significantly after 48 hours exposure to 4000 ng/mL DON from basolateral and aggravated during 72 hours toxin exposure. Along with this we also noticed a significant increase in nucleus area after 72 hours and 4000 ng/mL DON although not in a dose-dependent way. Some groups also reported a significant decrease in total cell numbers in a human K562 erythroleukaemia cell line in response to DON, although stating statistical significance already at 300 ng/mL DON for a 48 hour incubation [36]. However, these were immune cells that are known to be more sensitive to DON as compared to epithelial cell lines [37]. Interestingly, we could neither detect necrotic cell death as measured by LDH assay nor caspase 3 mediated apoptosis despite the drastic decrease in total cell count. This lack of LDH release in DON-treated intestinal epithelial cells (CaCo-2, IPEC-1) despite a marked decrease in TEER and cell reduction capacity (MTT assay) was earlier reported [22], [34]. In immune cells a significant increase in apoptotic cell death after 24 hours DON exposure (≥10 µg/mL) was given, but no effect on necrotic cell death [37]. The same study showed no impact on these parameters on the epithelial cell lines tested, BHK21 (kidney cells) and MH-22a (liver cells). Contradictory, some authors found a marked LDH release following DON exposure, but at rather high concentrations of 100 µM (≈30 µg/mL) for 48 hours (CaCo-2) and 30 µM (≈9 µg/mL) for 24 hours (HT-29-D4), respectively [23], [33].

One possible explanation for the lack of LDH release seen in our study could be that cells undergo anoikis rather than apoptosis. Anoikis represents a special form of programmed cell death where anchorage-dependent cells, such as epithelial cells, detach from their surrounding cell layer and matrix by severing bonds mediated by hemidesmosomes or integrins. This way the cell membrane of the dying cell is still intact and would not release LDH [38], [39].

Using flow cytometry we investigated the effect of mycotoxin exposure route on cell cycle. We observed a significant decrease in G0/G1 already after 48 h and an increase in preG1 phase after 72 hours basolateral DON application. Other groups reported a similar impact of the mycotoxin on cell cycle, in particular on cell cycle arrest in epithelial cells [40], [41]. The increase in preG1 phase, representing subdiploid, apoptotic cells [42], was also reported in IPEC-1 cells [43]. This increase points towards the apoptotic pathway rather than a cell cycle arrest. Caspase 3, one of the effector proteins of apoptosis, was not activated during the investigated time frame in our study, being somewhat contradictory to the data on cell cycle. However, caspase 3 was activated after 6, 8 and 12 hours of DON exposure in IPEC-1 or HT-29 cells grown on non-permeable plastic dishes [43], [44]. Probably either the signal for caspase 3 activation was too weak or the pathway of apoptosis was activated in a caspase 3 independent manner in cells growing on permeable inserts. Although caspase-dependent apoptosis seems to be a common type of cell death it became apparent that cells could also die when caspase function is blocked. This type of cell death is termed caspase-independent cell death (CICD) that occurs in response to mitochondrial outer membrane permeabilisation and disruption of mitochondrial morphology. In this process death receptor activation leads to so-called necroptosis (i.e. cell death) through the upregulation of phospholipase A_2_ (PLA2) activity that in turn increases oxidative stress or by effecting autophagy [45]. However, the question of caspase-independent cell death was not further pursued in our trial.

DNA synthesis was determined on single cell level using the thymidine-analogue BrdU. Although we observed a significant decrease in total cell count after 48 and 72 h of basolateral DON application, nearly all of those remaining cells were proliferating, possibly attempting to close the gaps in the IPEC-J2 layer. This resulted in a significant relative increase of proliferation compared to control. So far we can not decide whether this increased BrdU uptake is as primary DON effect or a secondary effect due to the cell death related gaps in the cell layer. Apical DON application elicited no response in DNA synthesis. This surprising result is in contrast to data reported in literature [46]. However, *in vitro* studies used predominantly BrdU-ELISA that provides information only for the entire cell population. Furthermore, relationships between the actual cell number and those of proliferating cells are rarely given and cells are cultivated on impermeable supports. One study used proliferating cell nuclear antigen (PCNA) as a single cell marker for proliferation, however this marker decreased in response to DON [47].

One striking aspect of former studies reporting on epithelial cells is that the cell layer was exposed to DON from the apical side in most cases. Most likely this originated from the idea that this apical exposure is the result of the oral intake of this mycotoxin whereby it will encounter the enterocytes from the luminal side. Furthermore, most assays, with the exception of TEER measurements were conducted on cells grown on non-permeable plastic or glass dishes. In our study we did see a surprising resilience of intestinal epithelial cells against DON when added to the apical side with virtually no effect in the applied assays. In contrast the basolateral application of the same mycotoxin concentration elicited a clear, dose-dependent response with impairment of the epithelial barrier integrity and an increase in apoptotic cell death, starting with quite lower DON concentrations than applied in other studies. As all experimental conditions were the same with the exception of route of toxin application we can assume that the cell support and the resulting changes in cell layer formation played a crucial role.

Other investigations reported that intestinal cells react indeed more sensitive to agents from the basolateral side as compared to apical exposure [48]. Interleukin-8 was significantly higher secreted by CaCo-2 cells grown on filters and exposed to *E. coli* from basolateral. This interleukin secretion was even amplified by DON, although this was only applied apically. The author’s explanation for this difference in sensitivity was the existence of toll-like receptor 5 (TLR-5) which is present on the basolateral side of the epithelial cell and in charge for the detection of bacterial flagellin [49]. Another study reported about the difference in impact of adenosine administered either from apical or basolateral to T84 colonic cells *in vitro* [50]. Ussing chamber experiments revealed a more potent effect of adenosine from basolateral on short-circuit current as well as on cellular cAMP. The authors explained these differences with distinct receptor proteins and density as well as different linkage to post-receptor signalling mechanisms. *In vivo*, a rapid absorption of DON in the upper small intestine of growing pigs, confined to the duodenum and the proximal jejunum, was found. Indicative for this rapid absorption were the fast serum peak concentrations (1.65 h±0.79 h) and the extremely low recovery of DON (1.5% of initial dose) after oral ingestion of the mycotoxin [15], [21]. Moreover, only 2.5% of the oral DON dose was excreted via faeces whereas the majority was excreted via the urinary tract. An efficient urinary excretion indicates a high gastro-intestinal absorption while a high faecal absorption might be due to an efficient biliary excretion or a lack of systemic absorption. As faecal elimination of DON in pigs was very low and systemic absorption very high we can deduce that a hepatic first pass effect of DON is negligible [15]. Although the toxin was in direct contact with the apical cell surface the authors could not see any alterations in terms of morphology and proliferation of epithelial cells. However, they reported marked effects in the mid-jejunum and ileum such as an increase in crypt depth and altered protein turnover, which in all likelihood could not have come in contact with substantial luminal amounts of DON. On one hand these *in vivo* data support our own results on the resilience of the apical cell surface towards DON. On the other hand it poses the question on how these epithelial changes could take place so distant from the actual site of absorption. One explanation would be that the mycotoxin enters the jejunal and ileal intestinal cells from the basolateral, i.e. blood stream side, after absorption in more proximal parts.

In conclusion, we could demonstrate that the response of the non-transformed, non-cancerous epithelial cell line IPEC-J2 to the mycotoxin deoxynivalenol differed dramatically depending on the route of toxin exposure. In particular, the basolateral domain of the intestinal barrier was significantly more vulnerable as compared to their apical counterpart. This result suggests a potential mechanism for the *in vivo* changes in intestinal barrier distal from the actual site of high apical DON exposure as discussed above. Moreover, this emphasises the importance of establishing a truly polarised cell layer when addressing physiological and pathophysiological questions of the intestinal epithelium *in vitro*.
